# Aids to Medical Diagnosis

**Published:** 1907-12

**Authors:** 


### Reviews of Books.

**Aids to Medical Diagnosis.** By Arthur Whiting, M.D. Pp. ix., 152. London: Baillière, Tindall & Cox. 1907.

This little book is intended for those who already possess a knowledge of systematic medicine. The plan adopted has been to start with the prominent symptoms in any given case, and then to separate the members of each group as clinical entities. The book therefore is essentially clinical in its aim, and the writer has succeeded in compressing a large amount of useful information into a small compass.

